# Clinical Utility And Diagnostic Accuracy of Faecal Calprotectin For IBD At First Presentation To Gastroenterology Services In Adults Aged 16–50 Years

**DOI:** 10.1016/j.crohns.2014.07.005

**Published:** 2014-12-18

**Authors:** Nicholas A. Kennedy, Annalie Clark, Andrew Walkden, Jeff C.W. Chang, Federica Fascí-Spurio, Martina Muscat, Brydon W. Gordon, Kathleen Kingstone, Jack Satsangi, Ian D.R. Arnott, Charlie W. Lees

**Affiliations:** ^a^Gastrointestinal Unit, Western General Hospital, Edinburgh, UK; ^b^Department of Clinical Biochemistry, Western General Hospital, Edinburgh, UK

**Keywords:** Crohn’s disease, Ulcerative colitis, Inflammatory bowel disease, Faecal calprotectin, Diagnostic test, Sensitivity

## Abstract

**Background:**

Distinguishing inflammatory bowel disease (IBD) from functional gastrointestinal (GI) disease remains an important issue for gastroenterologists and primary care physicians, and may be difficult on the basis of symptoms alone. Faecal calprotectin (FC) is a surrogate marker for intestinal inflammation but not cancer.

**Aim:**

This large retrospective study aimed to determine the most effective use of FC in patients aged 16–50 presenting with GI symptoms.

**Methods:**

FC results were obtained for patients presenting to the GI clinics in Edinburgh between 2005 and 2009 from the Edinburgh Faecal Calprotectin Registry containing FCs from >16,000 patients. Case notes were interrogated to identify demographics, subsequent investigations and diagnoses.

**Results:**

895 patients were included in the main analysis, 65% female and with a median age of 33 years. 10.2% were diagnosed with IBD, 7.3% with another GI condition associated with an abnormal GI tract and 63.2% had functional GI disease. Median FC in these three groups were 1251, 50 and 20 μg/g (p < 0.0001). On ROC analysis, the AUC for FC as a predictor of IBD vs. functional disease was 0.97. Using a threshold of ≥ 50 μg/g for IBD vs. functional disease yielded a sensitivity of 0.97, specificity of 0.74, positive predictive value of 0.37 and negative predictive value of 0.99. Combined with alarm symptoms, the sensitivity was 1.00.

**Conclusions:**

Implementation of FC in the initial diagnostic workup of young patients with GI symptoms, particularly those without alarm symptoms, is highly accurate in the exclusion of IBD, and can provide reassurance to patients and physicians.

## 1. Introduction

The relatively non-specific clinical manifestations of gastrointestinal disease can make it difficult for clinicians to distinguish between functional and organic intestinal disease, especially in patients presenting without rectal bleeding or systemic upset.^1,2^ The gold standard for identifying bowel inflammation, colonoscopy and histology, is an expensive and invasive procedure. Although attitudes to clinical targets have changed, endoscopic services are limited in many countries and a non-invasive tool to select individuals for early referral and investigations would enable the most cost effective use of resources.

Faecal calprotectin (FC), a 36.5 kDa calcium-binding cytosolic protein found in neutrophils, is increasingly being used in clinical practice as a surrogate marker for intestinal inflammation. FC correlates with faecal excretion of white cells and a number of studies have demonstrated that FC is significantly elevated in the stool of patients with active inflammatory bowel disease (IBD) compared to control groups.^3–6^ There is a large amount of existing literature relating to FC and its use in differentiating IBD and irritable bowel syndrome (IBS). However, the majority of these studies use data obtained from patients with a pre-existing diagnosis of IBD and IBS. Few studies assess the use of FC in undiagnosed populations; those that do analyze small sample sizes.^3,5–9^ FC is described by the British Society for Gastroenterology IBD guidelines as accurate in detecting colonic inflammation, and a NICE review was completed in October 2013.^10,11^ The systematic review that has been produced as part of this assessment reported that ‘calprotectin testing will lead to considerable savings to the NHS, as well as the avoidance of an unpleasant invasive procedure in people whose symptoms are due to IBS.’^12^

The current recommended upper limit of FC in the faeces of healthy individuals is 50 μg/g. A meta-analysis of adult patients has previously given sensitivity of 95% and a specificity of 91% when using a 50 μg/g cut-off threshold for differentiating IBD from healthy controls.^13^ A more recent meta-analysis of prospective studies using patients with suspected IBD found the pooled sensitivity and specificity of FC to be 93% and 96% respectively, although this analysis used studies with variable cut off values ranging from 24 to 150 μg/g.^14^ Importantly, FC is a poor test for colorectal cancer with a sensitivity and specificity of 36% and 71% respectively.^13^ However FC could potentially be used in clinical practice to identify young adult patients who require further invasive investigation to exclude intestinal inflammation. When used in the correct clinical scenario, with no alarm symptoms present, a negative FC result could be highly suggestive of an absence of organic gastrointestinal disease, thus usually avoiding the need for invasive investigation. Patients over the age of 50 years presenting with lower GI symptoms will require colonoscopy to exclude colorectal cancer.

Since 2005 a reliable FC assay has been available in the biochemistry department at the Western General Hospital, Edinburgh. More than 8000 assays had been performed by 2008. Our clinical practice has evolved to utilise FC values in two main areas. First, FC has been used to monitor disease activity in patients with established IBD; second, to exclude IBD in patients presenting to the out-patient clinic with lower GI upset. As confidence in the utility of FC has grown, we have tended in recent years to avoid invasive endoscopic and radiological investigation in young adult patients with a negative FC (< 50 μg/g) and no alarm symptoms.

This study aimed to determine the most effective use of FC in the diagnosis of GI disease in patients with no prior known GI disease, at the first presentation to GI services. We assessed how FC can be used as a non-invasive tool to aid referral to GI services, and how this improves cost effectiveness of resource allocation through reduction of unnecessary colonoscopies. Comparison was made against other serum markers to determine the optimal initial diagnostic workup of patients aged 16–50 years.

## 2. Methods

### 2.1 Patient population

Patient data were analysed from two large teaching hospitals within the same healthcare board (NHS Lothian): Western General Hospital (WGH), Edinburgh, and the Royal Infirmary Edinburgh (RIE). These were identified using the Edinburgh Faecal Calprotectin Register (EFCR), a record kept by the Biochemistry department at WGH. The EFCR contains the name, patient I.D., date of birth, referring hospital/department, and FC concentration for all of the samples analysed.

### 2.2 Derivation of cohort

**Figure 1.**
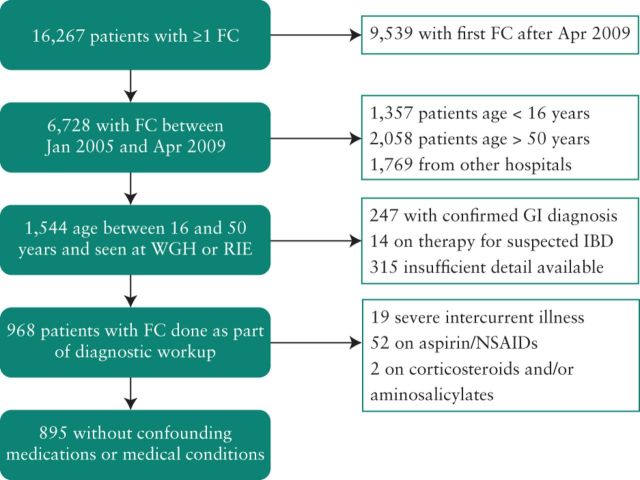
Derivation of the cohort.

The EFCR contains the data of 22,204 FC samples from 16,267 patients (Fig. 1). Patients were identified who had had their first FC between January 2005 and April 2009 to allow sufficient follow-up time to pick up cases of latent IBD. 1544 patients were aged 50 or under and had at least one sample originating from the WGH or RIE from the index period. Where multiple FC samples were listed for the same patient, the initial FC value from the patient's first presentation was included in all analyses. Subjects were excluded from the study if they had a confirmed GI diagnosis at time of sample (n = 247) or if they had already started treatment for presumed IBD (n = 14).

For the primary analysis, patients suffering from severe intercurrent illnesses (n = 19) were excluded as were patients receiving NSAID or aspirin therapy (n = 52), aminosalicylates and/or corticosteroids (n = 2), leaving 895 patients in the final cohort.

### 2.3 FC assay technique

FC was measured in a faecal extract using a standard enzyme-linked immunosorbent assay (ELISA) technique as previously described (Calpro AS, Norway).^15^ Faecal extract was added to a microtitre plate pre-coated with polyclonal antibodies to FC. Bound FC was detected using an alkaline phosphatase labelled human antibody to FC and quantified by comparison with a known standard preparation (numerical values given between 20 and 2500 μg/g). This assay was performed in the Department of Clinical Biochemistry at the Western General Hospital, Edinburgh. The reported assay precision for the calprotectin ELISA is a coefficient of variation (CV) of less than 6%. When including the faecal extraction step, the CV for the entire assay has been estimated to be less than 10% (unpublished data; 2014 email from Susan Walker, Department of Clinical Biochemistry, Western General Hospital, Edinburgh).

### 2.4 Data collection

Data was collected retrospectively by review of electronic patient records and recorded on a standardised data collection form. The electronic patient record system (Trak, Intersystems, Cambridge MA, USA) logs all patient contacts with secondary care (throughout NHS Lothian), including all endoscopic and radiological investigations, clinic appointments and hospital admissions. This data was then cross-referenced with other hospital electronic databases that store clinic letters and laboratory results in order to ensure the maximum retrieval and accuracy of data. Patients were followed up until at least three years after first presentation using Trak, ensuring all re-presentations and subsequent diagnoses were noted.

Parameters recorded were: age, gender, FC level and date of sample, presenting complaints (bloody diarrhoea, watery diarrhoea, rectal bleeding, constipation, abdominal pain, weight loss, flatulence/bloating, vomiting, dyspepsia, fatigue, possible extraintestinal manifestations, other), past medical history, family history (ulcerative colitis (UC), Crohn's disease (CD), IBD unclassified (IBDU), coeliac disease, colon cancer), smoking history (current at time of FC, ex- or never), drug history (including NSAIDs, antibiotics, laxatives, opioids, immunosuppresants, loperamide, aminosalicylates, acetaminophen, aspirin, corticosteroids), investigations performed (stool culture, colonoscopy, flexible sigmoidoscopy, upper GI endoscopy, abdominal ultrasound scan, abdominal X-ray, small bowel MRI, abdominal/pelvic CT, barium enema, barium follow-through, capsule endoscopy and radio-labelled white cell scan) and blood results (full blood count, liver function tests, erythrocyte sedimentation rate (ESR), C-reactive protein (CRP), albumin, hematinics (ferritin, vitamin B12 and serum folate), thyroid function tests, glucose, 7 alphahydroxycholestenone and anti-tissue transglutaminase IgA titre). Rectal bleeding, bloody diarrhoea, nocturnal symptoms, weight loss and anaemia were grouped as “alarm symptoms”. Where a laboratory test was reported as greater or less than a threshold, for statistical purposes it was assigned to one more or one less than the threshold respectively.

Any investigations performed were recorded as normal, abnormal or incomplete. “Abnormal” endoscopy findings included mucosal abnormalities, such as histologically proven malignancies and inflammation. The normal group includes those where no abnormalities were found as well as non-adenomatous polyps and haemorrhoids.

### 2.5 Diagnosis

Diagnosis was recorded as had been stated in the clinical notes. The Lennard-Jones criteria were used to diagnose IBD and the Montreal criteria to classify clinical phenotypes.^16,17^ The ROME III criteria were used to classify patients diagnosed with IBS.^18^ In cases where a diagnosis had not been recorded in the clinical notes, anonymised patient's notes were reviewed independently by two gastroenterologists blinded to the FC level (CWL and IDRA). Organic GI diagnoses were grouped as IBD, ‘abnormal gastrointestinal (GI) tract’ where a diagnosis would be expected to demonstrate a macroscopically abnormal GI tract and other GI where bidirectional endoscopy and capsule endoscopy would be expected to be normal. Details can be seen in Table S1.

Patients with a definitive organic diagnosis or who had undergone full colonoscopy (n = 467) were censored at the time of initial case note review. Those cases where an organic diagnosis was not made at the time of the FC or where no colonoscopy had been performed (n = 428) were reviewed in the last quarter of 2012 to ensure that no further cases of IBD or other significant GI pathology had been missed. Patients whose symptoms resolved spontaneously, who did not require further investigation and who did not re-present to hospital with GI symptoms were classified as ‘symptoms resolved.’ Those who were lost to follow-up without a definitive diagnosis were classed as ‘lost to follow-up’.

The main comparisons have been made in those with functional disease vs. those with IBD or another condition associated with an abnormal tract, since these are the patients in whom endoscopy would be a potentially useful test.

### 2.6 Cost analysis

Potential cost savings were calculated using 2012 tariff prices quoted by the Department of Health.^19^ One colonoscopy with biopsies in a patient aged 19 years or older was stated to cost £563, while a flexible sigmoidoscopy plus biopsy cost £360. The in-house processing cost of a single FC assay at WGH in 2008 was £24.47.

### 2.7 Statistical analysis

Statistical analyses of functional vs. organic groups and functional vs. IBD groups were performed. Medians and inter-quartile range are provided. Mann–Whitney U, Kruskal–Wallis, chi-squared and Fisher's exact tests were used to determine statistical significance. Receiver operating characteristic (ROC) curves were used to determine the best cut off point for FC when predicting organic disease and IBD. Comparison of area under the curve (AUC) was performed using the Delong and bootstrap methods. Positive predictive values (PPV) and negative predictive values (NPV) were calculated. Pre-test probabilities were calculated using all individuals regardless of FC concentration. Post-test probabilities were calculated with respect to different thresholds of FC.

A two-tailed p-value of less than 0.05 was considered significant. Confidence intervals for sensitivity and specificity were calculated using the method described by Newcombe with continuity correction.^20^ Confidence intervals for likelihood ratios were calculated using the method described by Simel et al.^21^ Statistical analyses were performed using R 3.0.1 (R Foundation for Statistical Computing, Vienna, Austria).

## 3. Results

### 3.1 Demographics

64.9% of patients were female, and the median age (interquartile range) at the time of FC was 33.1 years (25.6–40.7) (Table 1). 566/895 (63.2%) of patients were diagnosed with a functional disorder (Table 2). 91/895 (10.2%) were diagnosed with IBD, while a further 58 (7.3%) had conditions associated with an abnormal gastrointestinal tract. 63 patients (7.0%) had other miscellaneous gastrointestinal disorders. 78 patients (8.7%) did not have a final diagnosis, of whom 48 had complete symptomatic resolution and have not re-presented in ≥ 3 years, one has had further presentations with abdominal pain without a diagnosis while the remainder were lost to follow-up.

**Table 1.** Demographics of study population.

Variable		All n (%) or median (IQR)	Primary analysis cohort n (%) or median (IQR)
Sex	Female	627/968 (64.8%)	581/895 (64.9%)
Age at calprotectin/years		33.3 (25.7–41.0)	33.1 (25.6–40.7)
Smoking status at calprotectin	Current	204/641 (31.8%)	183/594 (30.8%)
	Ex	72/641 (11.2%)	68/594 (11.4%)
	Never	365/641 (56.9%)	343/594 (57.7%)
	Unknown	327/968 (33.8%)	301/895 (33.6%)
Drugs at calprotectin	NSAIDS	22/769 (2.9%)	0/701 (0.0%)
	Antibiotics	50/769 (6.5%)	0/701 (0.0%)
	Laxatives	16/769 (2.1%)	12/701 (1.7%)
	Opiates	39/769 (5.1%)	38/701 (5.4%)
	Immunosuppressants	82/769 (10.7%)	61/701 (8.7%)
	Loperamide	6/769 (0.8%)	2/701 (0.3%)
	Aminosalicylates	47/769 (6.1%)	42/701 (6.0%)
	Acetaminophen	1/769 (0.1%)	0/701 (0.0%)
	Aspirin	73/769 (9.5%)	52/701 (7.4%)
	Corticosteroids	2/769 (0.3%)	0/701 (0.0%)
	Unknown	199/968 (20.6%)	194/895 (21.7%)
Family history	None^a^	862/968 (89.0%)	794/895 (88.7%)
	UC	22/968 (2.3%)	21/895 (2.3%)
	CD	27/968 (2.8%)	26/895 (2.9%)
	IBDU	63/968 (6.5%)	60/895 (6.7%)
	Coeliac disease	11/968 (1.1%)	11/895 (1.2%)
	Colon cancer	14/968 (1.4%)	13/895 (1.5%)
Previous medical history	None^a^	920/968 (95.0%)	868/895 (97.0%)
	Inflammatory disease (non-gastrointestinal)	30/968 (3.1%)	24/895 (2.7%)
	Ankylosing spondylitis	30/968 (3.1%)	24/895 (2.7%)
	HIV	3/968 (0.3%)	2/895 (0.2%)
	Alcoholic liver disease	9/968 (0.9%)	0/895 (0.0%)
	Severe intercurrent illness	6/968 (0.6%)	1/895 (0.1%)

NSAIDS: non-steroidal anti-inflammatory drugs; UC: ulcerative colitis; CD: Crohn's disease; IBDU: inflammatory bowel disease unclassified.a

It has been assumed for this table that in the absence of any recorded previous medical history or family history in the patient records that there is none.

**Table 2.** Faecal calprotectin, age and time from calprotectin to diagnosis by diagnostic category.

Diagnosis category	n (%)	% female	Median age/years (IQR)		Median faecal calprotectin/μg/g (IQR)		Median time from calprotectin to diagnosis/days (IQR)	
Functional	566/895 (63.2%)	68.40%	32.7	(26.0–40.3)	20	(< 20–50.0)	95	(40–190)
IBD	91/895 (10.2%)	51.60%	29.8	(24.2–39.7)	1251	(532.5–2325.0)	7	(0–64)
Abnormal GI tract	65/895 (7.3%)	53.80%	37.7	(26.1–44.4)	50	(20.0–145.0)	92	(41–206)
Other GI	63/895 (7.0%)	65.10%	35	(27.0–42.8)	20	(< 20–70.0)	92	(35–153)
Other organic	32/895 (3.6%)	68.80%	31	(25.3–41.4)	22.5	(< 20–86.2)	106	(34–192)
Lost to Fup	29/895 (3.2%)	62.10%	35.8	(26.5–43.2)	135	(35.0–325.0)		
None	1/895 (0.1%)	100.00%	20.8		1825			
Symptoms resolved — no GI pathology	48/895 (5.4%)	62.50%	34.3	(25.3–42.7)	35	(< 20–76.2)		

### 3.2 FC and demographic variables

FC was not significantly associated with age (p = 0.21), sex (p = 0.18) or current smoking (p = 0.80).

### 3.3 FC and other clinical parameters assessed by final diagnosis

**Figure 2.**
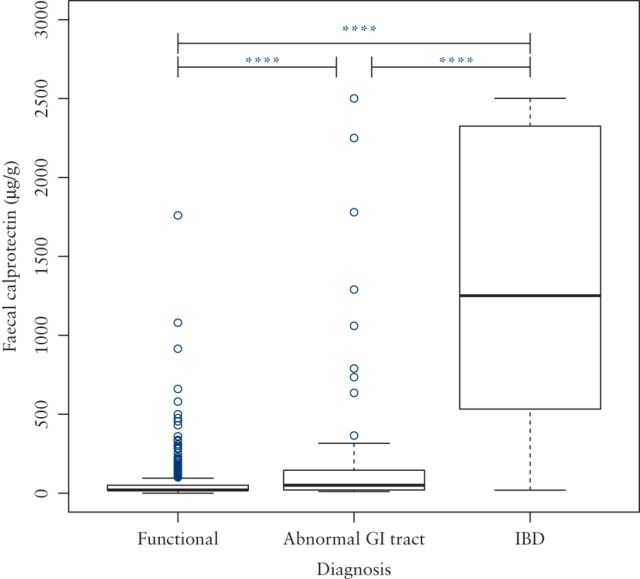
Box plot showing difference in faecal calprotectin between patients with functional diagnoses and those with IBD and other conditions associated with an abnormal GI tract.

FC was significantly higher in patients diagnosed with IBD (median FC 1251 μg/g, IQR 532–2325 μg/g) than those with other conditions associated with an abnormal gastrointestinal tract (median FC 50 μg/g, IQR 20–145 μg/g) or with a functional diagnosis (median FC 20 μg/g, IQR < 20–50 μg/g) (p ≤ 0.0001 in each case, see Fig. 2).

### 3.4 FC in patients taking non-steroidal anti-inflammatories (NSAIDs)

Patients taking NSAIDs or aspirin were excluded from the primary analysis. In patients with a functional diagnosis, the FC was significantly higher in those taking NSAIDs or aspirin when compared with those on neither drug (median FC 52 μg/g [IQR < 20–181 μg/g] vs. 20 μg/g [IQR < 20–50 μg/g], p = 0.001).

### 3.5 FC in patients with IBD

Of the 91 patients ultimately diagnosed with IBD, 40 (44%) had CD, 41 (45%) had UC and 10 (11%) had IBDU. There was no significant difference in FC between the three subtypes of IBD (p = 0.56). Within the group with CD, there were 10 (25%) with L1 (ileal) disease, including one patient with L1 + 4, 18 (45%) with L2 (colonic) disease and 12 (30%) with L3 (ileocolonic) disease. FC was significantly higher in those with L2 or L3 disease, with a median (IQR) of 1280 (714–2295) μg/g than in those with L1 disease where median (IQR) FC was 495 (288–822) μg/g (p = 0.009) (Supplementary Fig. 1).

Within the group with UC, there were 3 (7%) with E1 disease (proctitis), 12 (29%) with E2 disease (left-sided colitis) and 21 (51%) with E3 disease (extensive colitis). In the remaining 5 patients, the disease extended beyond the point of insertion of the sigmoidoscope and complete staging of extent was not achieved during the initial diagnostic period. There was no significant difference in FC by disease extent when those without complete staging were excluded (p = 0.25 by Kruskal–Wallis test; Supplementary Fig. 2).

Across all patients diagnosed with IBD, there was no significant association between time to diagnosis and faecal calprotectin.

### 3.6 Diagnostic accuracy of FC compared to other clinical parameters

**Figure 3.**
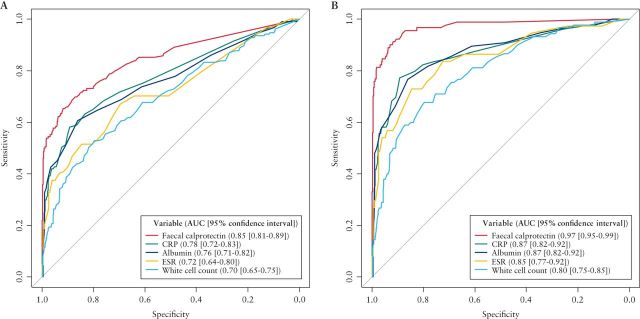
Receiver operating characteristic curves for calprotectin, CRP, albumin, ESR and white cell count as predictors of inflammatory bowel disease (IBD) or abnormal gastrointestinal tract versus functional disease (A) and IBD versus functional disease (B).

Receiver operating characteristic (ROC) analysis revealed an area under the curve (AUC) for FC of 0.85 for prediction of conditions with an abnormal GI tract (including IBD) vs. functional disease, and 0.97 for prediction of IBD vs. functional disease (Fig. 3). This was significantly higher than that seen for CRP, albumin, ESR or white cell count in both cases (p < 0.001 for all comparisons). The sensitivities, specificities, and positive and negative predictive values for faecal calprotectin can be seen in Table 3 at different thresholds. Summaries of the number of available tests, medians and interquartile ranges for each parameter can be seen in Supplementary Table S2.

**Table 3.** Diagnostic accuracy of fecal calprotectin at different thresholds PPV: positive predictive value; NPV: negative predictive value; PLR: positive likelihood ratio; CI: confidence interval.

Threshold fecal calprotectin (μg/g)	Sensitivity (95% CI)		Specificity (95% CI)		PPV (95% CI)		NPV (95% CI)		PLR (95% CI)	
*A: Inflammatory bowel disease (IBD) or abnormal GI tract vs. functional disease*										
20	0.89	(0.83–0.93)	0.49	(0.44–0.53)	0.32	(0.28–0.37)	0.94	(0.91–0.96)	1.73	(1.57–1.91)
50	0.79	(0.71–0.85)	0.74	(0.70–0.77)	0.45	(0.39–0.52)	0.93	(0.90–0.95)	3.02	(2.57–3.54)
70	0.73	(0.65–0.80)	0.80	(0.76–0.83)	0.50	(0.44–0.57)	0.92	(0.89–0.94)	3.66	(3.03–4.43)
100	0.70	(0.62–0.77)	0.87	(0.84–0.90)	0.60	(0.52–0.67)	0.91	(0.89–0.93)	5.42	(4.27–6.87)
*B: IBD vs. functional disease*										
20	0.99	(0.93–1.00)	0.49	(0.44–0.53)	0.24	(0.20–0.28)	1.00	(0.98–1.00)	1.92	(1.77–2.09)
50	0.97	(0.90–0.99)	0.74	(0.70–0.77)	0.37	(0.31–0.44)	0.99	(0.98–1.00)	3.70	(3.20–4.27)
70	0.97	(0.90–0.99)	0.80	(0.76–0.83)	0.44	(0.37–0.51)	0.99	(0.98–1.00)	4.84	(4.09–5.74)
100	0.96	(0.89–0.99)	0.87	(0.84–0.90)	0.54	(0.46–0.62)	0.99	(0.98–1.00)	7.41	(5.96–9.22)

### 3.7 Synergistic effect of FC sampling and alarm symptoms

Alarm symptoms were present in 25% (140/566) of those ultimately diagnosed with functional disease, 86% (78/91) of those diagnosed with IBD and 54% (35/65) of those diagnosed with another condition associated with an abnormal GI tract (p < 0.0001). The positive predictive value of alarm symptoms for IBD or an abnormal GI tract vs. functional disease was 0.45 (95% CI 0.38–0.51), and the negative predictive value was 0.91 (0.88–0.93) with a sensitivity of 0.72 (0.65–0.79) and specificity of 0.75 (0.71–0.79). For prediction of IBD vs. functional disease, the PPV was 0.36 (0.29–0.43) and NPV was 0.97 (0.95–0.98), with a sensitivity of 0.86 (0.76–0.92) and specificity of 0.75 (0.71–0.79).

As can be seen in Table 4, FC is helpful in improving the prediction of an abnormal GI tract or IBD compared with alarm symptoms alone. Within the cohort with functional disease or an abnormal GI tract, none of the 329 patients with no alarm symptoms and a FC of < 50 μg/g was found to have IBD, while 11/36 (31%) of patients with no alarm symptoms and a FC of ≥ 200 were found to have IBD.

**Table 4.** Pre- and post-test probabilities when combining alarm symptoms and fecal calprotectin.

	Pre-test probability	Post-test probability for different values of fecal calprotectin (μg/g)				
		< 20	20–49	50–99	100–199	200 +
A: inflammatory bowel disease (IBD) or abnormal GI tract vs. functional disease						
Alarm symptoms	0.45	0.15	0.18	0.24	0.50	0.91
No alarm symptoms	0.09	0.03	0.06	0.12	0.20	0.41
B: IBD vs. functional disease						
Alarm symptoms	0.36	0.02	0.05	0.05	0.41	0.89
No alarm symptoms	0.04	0.00	0.00	0.00	0.06	0.33

Thirteen patients had no alarm symptoms and a FC of < 50 μg/g, but were found to have a disease associated with an abnormal GI tract. These were 1 case of appendicitis, 1 coeliac disease, 3 with colonic adenomatous polyps, 1 with diverticulosis, 3 with GI infections (1 *Fasciola hepatica*, 1 giardiasis, 1 presumed infection with response to metronidazole), 2 with gastro-oesophageal reflux disease and 2 non-specific bowel inflammation. One of these patients with non-specific bowel inflammation was initially thought to have CD but had non-specific changes on her index colonoscopic biopsies and subsequently normal colonoscopy and biopsies.

### 3.8 Multivariable analysis

Multiple logistic regression analysis of predictors of IBD vs. functional disease showed that elevated FC, elevated CRP, male sex, alarm symptoms and albumin were independently significant. Age at FC and white cell count were not (Table 5).

**Table 5.** Multiple logistic regression of predictors of inflammatory bowel disease vs. functional disease. CRP: C-reactive protein.

Variable	Odds ratio (95% CI)	P
Fecal calprotectin≥50μg/g	65.3 (12.1–351.5)	1.1×10^−6^
Alarm symptoms	19.5 (7.9–127.5)	3.0×10^−6^
Albumin<40g/L	18.7 (4.1–85.4)	3.0×10^−5^
Male sex	14.1 (3.8–52.2)	7.0×10^−5^
CRP≥5g/L	6.9 (2.0–23.7)	0.002
Age at calprotectin		>0.05
White cell count >11×10^9^/L		>0.05

Comparing different strategies of investigation (Table 6) demonstrated that FC alone provided the optimum specificity for both IBD vs. functional disease and IBD or abnormal GI tract vs. functional disease. The optimal combination of sensitivity and specificity was attained using the approach of alarm symptoms or FC ≥ 50 μg/g. Sensitivity and specificity for IBD vs. functional disease were 1.00 and 0.54 with this strategy, while for IBD or abnormal GI tract vs. functional disease they were 0.96 and 0.55. Adding CRP to this combination had minimal effect on sensitivity, while reducing specificity.

**Table 6.** Comparison of different strategies for identifying IBD or abnormal gastrointestinal (GI) tract vs. functional disease. IBD: inflammatory bowel disease; CRP: C-reactive protein.

Strategy	IBD vs. functional		IBD or abnormal GI tract vs. functional	
	Sensitivity	Specificity	Sensitivity	Specificity
Alarm symptoms only	0.85	0.73	0.76	0.74
CRP≥5g/L only	0.85	0.70	0.71	0.70
Faecal calprotectin (FC)≥50μg/g only	0.97	0.74	0.86	0.75
Alarm symptoms or CRP≥5g/L	0.99	0.50	0.89	0.51
Alarm symptoms or FC≥50μg/g	1.00	0.54	0.96	0.55
Alarm symptoms or CRP≥5g/L or FC≥50μg/g	1.00	0.39	0.97	0.39
Alarm symptoms or (CRP≥5g/L and FC≥50μg/g)	0.99	0.65	0.88	0.67

### 3.9 Low FC in patients diagnosed with inflammatory bowel disease

Three patients had a low FC (< 50 μg/g) and were diagnosed with inflammatory bowel disease. All three had alarm symptoms (two had blood in their stool and one had weight loss). Two of these patients were diagnosed with ulcerative proctitis which has not extended further in > 4 years of follow-up. One had mild terminal ileal CD with no subsequent progression.

### 3.10 Cost effectiveness of FC: reducing the number of invasive investigations

Between 2005 and 2008, our practice evolved with increasing use of FC and reduction in the percentage of these patients subsequently undergoing invasive investigation. In the 2005, 63 patients underwent stool analysis for FC with 84.1% of them undergoing either sigmoidoscopy or colonoscopy. In 2008, 409 patients had stool sent for FC with 56.7% subsequently undergoing invasive investigation (Table S3).

Over the study period, 581/895 (64.9%) patients presented without alarm symptoms. 395 of these (68.0%) had a FC of < 50 μg/g. 150 of these patients (38%) had a subsequent colonoscopy and 50 (13%) a flexible sigmoidoscopy, identifying incidental adenomatous polyps in 3 patients and no other significant pathology. If the low FC had been used to triage these patients to a non-invasive approach, this would have saved £88,233 over that time period.

## 4. Discussion

This study uses the largest, ‘real-world’ population of undiagnosed patients to determine the best way of using FC at first presentation to the GI clinic to differentiate non-invasively between organic and functional disease. This allows identification of those in need of efficient and effective further investigation. Incorporating FC into the standard work-up of patients presenting with lower GI symptoms may potentially relieve pressure on hospital services by identifying patients who can be managed solely in primary care.

Our findings corroborate existing data showing that FC reliably distinguishes between patients with functional disease and IBD. Von Roon et al.'s meta-analysis of adult patients demonstrated a sensitivity of 95% and specificity of 91% when using a 50 μg/g cut-off point for differentiating IBD patients from healthy controls.^13^ At the same cut-off, our study found 95% sensitivity but only 75% specificity. This agreement in sensitivity reinforces the diagnostic ability of FC in identifying patients with IBD in a large cohort of patients. The lower specificity seen in our study may be due to the patient population used, all of which have presented to services with GI symptoms, unlike the healthy control population used by Von Roon et al. Van Rheenen et al's more recent meta-analysis of six adult studies found a pooled sensitivity and specificity of 93% and 96% respectively.^22^ However, inconsistent FC thresholds were used in these six studies, with 47.7% of included patients analysed using a cut-off greater than 100 μg/g, and this may have influenced the specificity.

Both ESR and CRP are markers that are commonly used to identify systemic inflammation in patients with IBD-like symptoms. In accordance with previous research, we show CRP and ESR are raised in patients with organic disease and IBD.^4,23^ ROC analysis demonstrates however, that FC is superior to CRP and ESR in the diagnosis of IBD — a finding that agrees with the recent economic report produced by the NHS Centre for Evidence Based Purchasing.^24^ Furthermore, we demonstrate that the NPV of FC in patients presenting with no alarm symptoms is superior to the NPV of CRP for both organic GI disease and IBD. Cost savings could be made by solely checking FC in patients presenting with lower GI symptoms, rather than checking CRP and ESR in these patients

One of the most clinically relevant findings from our data is the NPV for IBD of 99.0% when a FC threshold of 50 μg/g is used. When FC less than 50 μg/g is combined with the absence of alarm symptoms, NPV is 100.0% for IBD. This allows the exclusion of IBD from the differential diagnosis of these patients. Furthermore, in patients meeting these criteria, NPV for any GI tract abnormality is 96.1%. Of the 13 patients with no alarm symptoms and FC less than 50 μg/g who had a diagnosis of abnormal GI tract, colonoscopy was helpful in only four patients and these (diverticular disease and colonic polyps) were likely incidental findings. Clinicians can therefore be reassured that referral for colonoscopy will not identify severe organic disease in patients in whom no abnormalities are found in initial investigations. This finding could potentially be applied to a primary care scenario and aid selection of patients for colonoscopy.

With the Department of Health pricing a single colonoscopy in adults at £563 there is great potential for FC to aid more cost-effective decision making with regard to further investigation.^19^ Von Rheenen et al.'s meta-analysis demonstrated that screening with FC could reduce unnecessary colonoscopies by 67% in those suspected of having IBD.^22^ Similar results were documented by Mindemark and colleagues, with a reduction of colonoscopies by 50% using the FC cut off of < 50 μg/g and 67% using a FC cut off of < 100 μg/g.^25^ During the study period of the present study, if patients with a FC < 50 μg/g and no alarm symptoms had not undergone lower GI endoscopy there could have been 150 fewer colonoscopies and 50 fewer flexible sigmoidoscopies. Our data reflects real world practice, with proportionally fewer patients being investigated by colonoscopy as our knowledge and experience of FC increased. A reducing trend in the numbers of those patients investigated with colonoscopy can clearly be seen as the number of FC assays received by the labs increase over the three years. The number of potential colonoscopies saved quoted above may even be more than this had our unit not been internally evaluating FC's use in clinical practice. Furthermore, the numbers we have analyzed only include patients who attended the GI clinic and had a FC sample sent. These findings could be applied to all patients who attend the GI clinic with lower GI symptoms, potentially reducing further the number of colonoscopies and resulting in even greater cost savings. It is important to take into consideration that this study uses patients referred to hospital GI services, and by virtue of this the spectrum of symptoms seen in this population is more severe when compared to all the patients presenting to GPs with GI symptoms. In primary care, FC could identify the small numbers of patients with IBD, whilst excluding its presence in a large number of patients presenting with GI symptoms. Not only could this streamline the referral of appropriate patients to hospital, but it will also reduce the number of unnecessary referrals and invasive investigations. This does, however, require detailed pilot testing before any formal recommendations about the roll-out of FC into primary care can be made. Moreover, it is important that FC is used in the context of a defined protocol to ensure that it does not delay referral of patients with alarm symptoms and that consideration is given to possible false positive tests from aspirin and non-steroidal inflammatory drugs.

One of the strengths of this study is that all individuals without a definitive diagnosis or in whom a functional diagnosis had been made without colonoscopy were re-reviewed three years later to identify any possible latent cases of IBD or other GI disease.

This study clarifies important, clinically relevant information about FC. Awareness of the high negative predictive value of FC allows clinicians to effectively exclude IBD as a cause for gastrointestinal symptoms in patients with FC levels under 50 μg/g. FC can thus be used as an adjunct to other presenting complaints and investigations, allowing the risk stratification of patients presenting with gastrointestinal symptoms in a cost-effective manner.

## Statement of interests

NAK is funded by a research training fellowship from the Wellcome Trust[grant 097943] and has had financial support to attend meetings from Warner Chillcott, Shire, MSD and Norgine. He has had speaker fees from MSD and Warner Chilcott.

JS has had research funding from Abbvie, speaker fees from Ferring and travel support from Shire.

IDRA has been on advisory boards for MSD, Hospira and P&G.

CWL has been on advisory boards for and had lecture fees from Abbvie, Hospira, MSD, Vifor, Pharmacosmos and P&G.

## Abbreviations

AUC: area under the curve
CD: Crohn's disease
CRP: C-reactive protein
ESR: erythrocyte sedimentation rate
FC: faecal calprotectin
IBD: inflammatory bowel disease
IBDU: inflammatory bowel disease unclassified
NHS: National Health Service
NICE: National Institute of Clinical Excellence
NPV: negative predictive value
PPV: positive predictive value
ROC: receiver operating curve
UC: ulcerative colitis

## Appendix A Supplementary data

Supplementary data to this article can be found online at http://dx.doi.org/10.1016/j.crohns.2014.07.005.
